# Cytosolic Free *N*-Glycans Are Retro-Transported Into the Endoplasmic Reticulum in Plant Cells

**DOI:** 10.3389/fpls.2020.610124

**Published:** 2021-01-18

**Authors:** Makoto Katsube, Natsuki Ebara, Megumi Maeda, Yoshinobu Kimura

**Affiliations:** Department of Biofunctional Chemistry, Graduate School of Environmental and Life Science, Okayama University, Okayama, Japan

**Keywords:** free N-glycans, ER-associated degradation, peptide:N-glycanase, endo-β-N-acetylglucosaminidase, plant glycoproteins

## Abstract

During endoplasmic reticulum (ER)-associated degradation, free *N*-glycans (FNGs) are produced from misfolded nascent glycoproteins *via* the combination of the cytosolic peptide *N*-glycanase (cPNGase) and endo-β-*N*-acetylglucosaminidase (ENGase) in the plant cytosol. The resulting high-mannose type (HMT)-FNGs, which carry one GlcNAc residue at the reducing end (GN1-FNGs), are ubiquitously found in developing plant cells. In a previous study, we found that HMT-FNGs assisted in protein folding and inhibited β-amyloid fibril formation, suggesting a possible biofunction of FNGs involved in the protein folding system. However, whether these HMT-FNGs occur in the ER, an organelle involved in protein folding, remained unclear. On the contrary, we also reported the presence of plant complex type (PCT)-GN1-FNGs, which carry the Lewis^a^ epitope at the non-reducing end, indicating that these FNGs had been fully processed in the Golgi apparatus. Since plant ENGase was active toward HMT-*N*-glycans but not PCT-*N*-glycans that carry β1-2xylosyl and/or α1-3 fucosyl residue(s), these PCT-GN1-FNGs did not appear to be produced from fully processed glycoproteins that harbored PCT-*N*-glycans *via* ENGase activity. Interestingly, PCT-GN1-FNGs were found in the extracellular space, suggesting that HMT-GN1-FNGs formed in the cytosol might be transported back to the ER and processed in the Golgi apparatus through the protein secretion pathway. As the first step in elucidating the production mechanism of PCT-GN1-FNGs, we analyzed the structures of free oligosaccharides in plant microsomes and proved that HMT-FNGs (Man_9-7_GlcNAc_1_ and Man_9-8_GlcNAc_2_) could be found in microsomes, which almost consist of the ER compartments.

## Introduction

Free *N*-glycans (FNGs), which are related to asparagine-linked glycoproteins, are widely found in various eukaryotes, including yeast, plants, and animals. These FNGs can be classified into two types, GN1 and GN2, based on the reducing terminal structure; GN1-FNGs have one GlcNAc residue, whereas GN2-FNGs have GlcNAcβ1-4GlcNAc (the *N,N'*-diacetyl chitobiosyl unit). Regarding the protein quality control system in the endoplasmic reticulum (ER) in both animal and plant cells, it has been believed that misfolded *N*-glycoproteins that harbor high-mannose type (HMT)-*N*-glycans are transported through the dislocone complex (0s9-Sel1-HRAD1 complex) into the cytosol for proteasomal degradation or ER-associated degradation (ERAD; Suzuki and Funakoshi, 2006; Hosokawa et al., 2009, 2010; Abei et al., 2010; Hüttner and Strasser, 2012; Suzuki and Harada, 2014; Suzuki, 2015). Before proteolytic degradation, these misfolded *N*-glycoproteins are first de-*N*-glycosylated by cytosolic PNGase (cPNGase), and the resulting GN2-FNGs are then further processed into GN1-FNGs by endo-β-*N*-acetylglucosaminidase (ENGase), which is highly specific for HMT-GN2-FNGs. It has been believed that the resulting HMT-GN1-FNGs are further degraded by cytosolic α-mannosidase (α-Man’ase) and finally transported to the lysosome, where the FNGs are degraded to monosaccharides, in animal cells (Suzuki et al., 2006; Kato et al., 2011; Wang and Suzuki, 2013). The fate of HMT-GN1-FNGs formed in plant cytosol, in sharp contrast to those formed in mammalian cytosol, remains to be clarified, since cytosolic α-Man’ase has not been found and no orthologous gene of the animal cytosolic α-Man’ase has been identified to date. This indicates that the HMT-FNGs produced during ERAD may be metabolized *via* a slightly different pathway in animal cells.

As for complex type FNGs (CT-FNGs), both GN1-FNGs and GN2-FNGs have been found in both mammalian and plant cells or in their extracellular spaces (Priem et al., 1993; Faugeron et al., 1997a,b; Kimura et al., 1997, 2000; Ohashi et al., 1999; Ishizuka et al., 2008; Nakamura et al., 2008; Maeda et al., 2010, 2017; Iwatsukasa et al., 2013; Wang et al., 2015; Seino et al., 2016), and the structural features of these FNGs clearly suggest that these *N*-glycans had been modified or processed in the Golgi apparatus. It has been proposed that animal complex type (ACT)-GN2-FNGs are formed from HMT-GN2-FNGs, which are produced from dolichol-linked oligosaccharides as byproducts of the reaction involving the transfer of the glycan moiety (GlcNAcMan9GlcNAc2) to specific Asn residues in nascent polypeptides in the ER by oligosaccharyltransferase (OST) in mammalian cells (Harada et al., 2015). They have postulated that these HMT-GN2-FNGs, along with well-folded glycoproteins, are possibly transported and modified in the Golgi apparatus. Interestingly, extracellular ACT-FNGs are almost exclusively of the GN2 type, and this observation suggests that the mechanism of GN1-FNG generation may be slightly different from that of GN2-FNG generation.

In previous reports (Maeda et al., 2010, 2017), the plant complex type (PCT)-GN1-FNGs, which carry the Le^a^ epitope [Galβ1-3(Fucα1-4)GlcNAc], were found in the culture broth of rice cells or the crude extract of a freshwater plant, *Egeria densa*. Since it has already been confirmed that plant ENGase is almost inactive toward typical plant-specific *N*-glycans, such as M3FX, GN2M3FX, and GN2M3X (Kimura et al., 1998, 2002; Maeda et al., 2017), these PCT-GN1-FNGs did not appear to be generated from *N*-glycopeptides or *N*-glycoproteins that harbored PCT-*N*-glycans by ENGase activity during turnover of the function-lost glycoproteins. Therefore, we proposed that these PCT-GN1-FNGs might have originated from HMT-GN1-FNGs produced from misfolded glycoproteins *via* the combination of cPNGase and ENGase in the cytosol during ERAD (Maeda et al., 2010; Maeda and Kimura, 2014). We proposed the following hypothesis regarding the formation of PCT-GN1-FNGs from HMT-GN1-FNGs: during the first stage, HMT-GN1-FNGs produced from misfolded glycoproteins might be transported back to the ER through an unidentified transporter; then, these HMT-FNGs, along with well-formed glycoproteins, might be transported to the Golgi apparatus. During the second stage, these HMT-GN1-FNGs might be processed into PCT-GN1-FNGs via concerted reactions mediated by Golgi-glycosidases and transferases, along with the *N*-glycans of secreted-type glycoproteins. Finally, the resulting PCT-GN1-FNGs might be secreted into the extracellular space. The fact that these PCT-GN1-FNGs have been found in the culture broth, but not in rice cells (Maeda et al., 2010), appears to support this hypothesis. Furthermore, we found that HMT-FNGs assisted in protein folding and inhibited β-amyloid fibril formation, suggesting a possible biofunction of FNGs in the protein folding system (Tanaka et al., 2015). However, whether these HMT-FNGs occurred in the ER, an organelle involved in the folding of secreted-type glycoproteins, remained unclear.

As the first step to prove that HMT-FNGs (GN1 and/or GN2) occur in the ER, we prepared plant microsomes from pumpkin hypocotyls (Kimura et al., 2002) and analyzed the structural features of free oligosaccharides in the microsomes in this study. We found that HMT-FNGs (GN1 and GN2) occurred in the microsomes that were mainly contained in the ER compartments. These results suggested that GN1-FNGs generated from misfolded glycoproteins *via* the combination of cPNGase and ENGase were retro-transported from the cytosol to the ER by a putative transporter specific for FNGs.

## Materials and Methods

### Materials

A Cosmosil 5C18-AR column (0.60 × 25 cm) was purchased from NacalaiTesque, Inc. (Kyoto, Japan), and a Shodex Asahipak NH2P-50 column (0.46 × 25 cm) was purchased from Showa Denko Co. (Tokyo, Japan). Man_9-5_GlcNAc_1_-PA and Man_9-5_GlcNAc_1_-PA were prepared as described in previous reports (Kimura et al., 2000; Kimura and Matsuo, 2000). α-1,2-Man’ase from *Aspergillus saitoi* was purchased from ProZyme, Inc. (Hayward, CA, United States). Swainsonine, deoxymannojirimycin, deoxynojirimycin, and Jack bean β-GlcNAc’ase were purchased from Sigma-Aldrich (St. Louis, MO, United States), and Endo-H was purchased from Promega (Madison, WI, United States). Glc3Man9GlcNAc2-PA and Glc2Man9GlcNAc2-PA were purchased from Masuda Chemical Industries Co. (Kagawa, Japan).

### Reverse-Phase-High Performance Liquid Chromatography and Size Fractionation-HPLC

Fluorescence-labeled oligosaccharides were separated by high performance liquid chromatography (HPLC) using a Jasco 2080-PU HPLC system equipped with a Jasco 920-FP Intelligent Spectrofluorometer (excitation 310 nm and emission 380 nm; Jasco, Tokyo, Japan). For reverse-phase (RP)-HPLC using a Cosmosil 5C18-AR-II (4.6 × 250 mm), the PA-sugar chains were eluted by increasing the acetonitrile concentration in 0.02% trifluoroacetic acid (TFA) linearly from 0 to 7% at a flow rate of 1.2 ml/min. For size fractionation (SF)-HPLC using a Shodex Asahipak NH2P-50 4E (4.6 × 250 mm), the PA-sugar chains were eluted by increasing the water content of the water-acetonitrile mixture from 26 to 50% linearly at a flow rate of 0.7 ml/min.

### ENGase Assay

ENGase activity was assayed using M6B as a substrate and M3FX [Manα1-6(Manα1-3)(Xylβ1-2)Manβ1-4GlcNAcβ1-4(Fucα1-3)GlcNAc-PA] as an internal standard, as described in our previous paper (Kimura et al., 2011). Briefly, an enzyme solution (100 μl) was mixed with M6B and M3FX (approximately 100 pmol) in 0.1 M MES buffer (44 μl, pH 6.5) containing 5 mM swainsonine (3 μl) and 5 mM deoxymannojirimycin (3 μl). After incubation at 37°C overnight, the reaction was stopped by heating at 100°C for 3 min. After centrifugation, an aliquot (50 μl) of the resulting supernatant was analyzed by RP-HPLC using the Cosmosil 5C18-AR column. The PA-sugar chains (M6B, M3FX, and PA-GlcNAc) were eluted as described above. The substrate specificity of pumpkin ENGase has been reported using various pyridylaminated *N*-glycans in our previous report (Kimura et al., 2002).

### ER α-Glucosidase I and II Assay

Endoplasmic reticulum α-glucosidase I and II activities were assayed using Glc_3_Man_9_GlcNAc_1_-PA (G3M9’) and Glc_2_Man_9_GlcNAc_1_-PA (G2M9’) as substrates, respectively. G3M9’ and G2M9’ were prepared from Glc_3_Man_9_GlcNAc_2_-PA and Glc_2_Man_9_GlcNAc_2_-PA, respectively, by Endo-H digestion followed by pyridylamination, as described below. An enzyme solution (100 μl) was mixed with G3M9’ and G2M9’ (approximately 50 pmol) in 0.1 M MES buffer (44 μl, pH 6.5) containing 5 mM swainsonine (3 μl), 5 mM deoxymannojirimycin (3 μl), and 5 mM deoxynojirimycin (3 μl). After incubation at 37°C overnight, the reaction was stopped by heating at 100°C for 3 min. The digested substrates were analyzed by SF-HPLC using the Shodex NH2P-50 4E column.

### α-1,2-Mannosidase Digestion

PA-sugar chains obtained from the pumpkin microsomal fraction were incubated with *Aspergillus* α-1,2-Man’ase (100 μU) in 0.1 M Na-acetate buffer (pH 5.0) at 37°C overnight. The reactions were stopped by boiling the mixtures for 3 min, and a part of each digested substrates were analyzed by SF-HPLC using the Shodex Asahipak NH2P-50 4E column.

### Preparation of Microsomes From Pumpkin Hypocotyls

**Figure 1:**
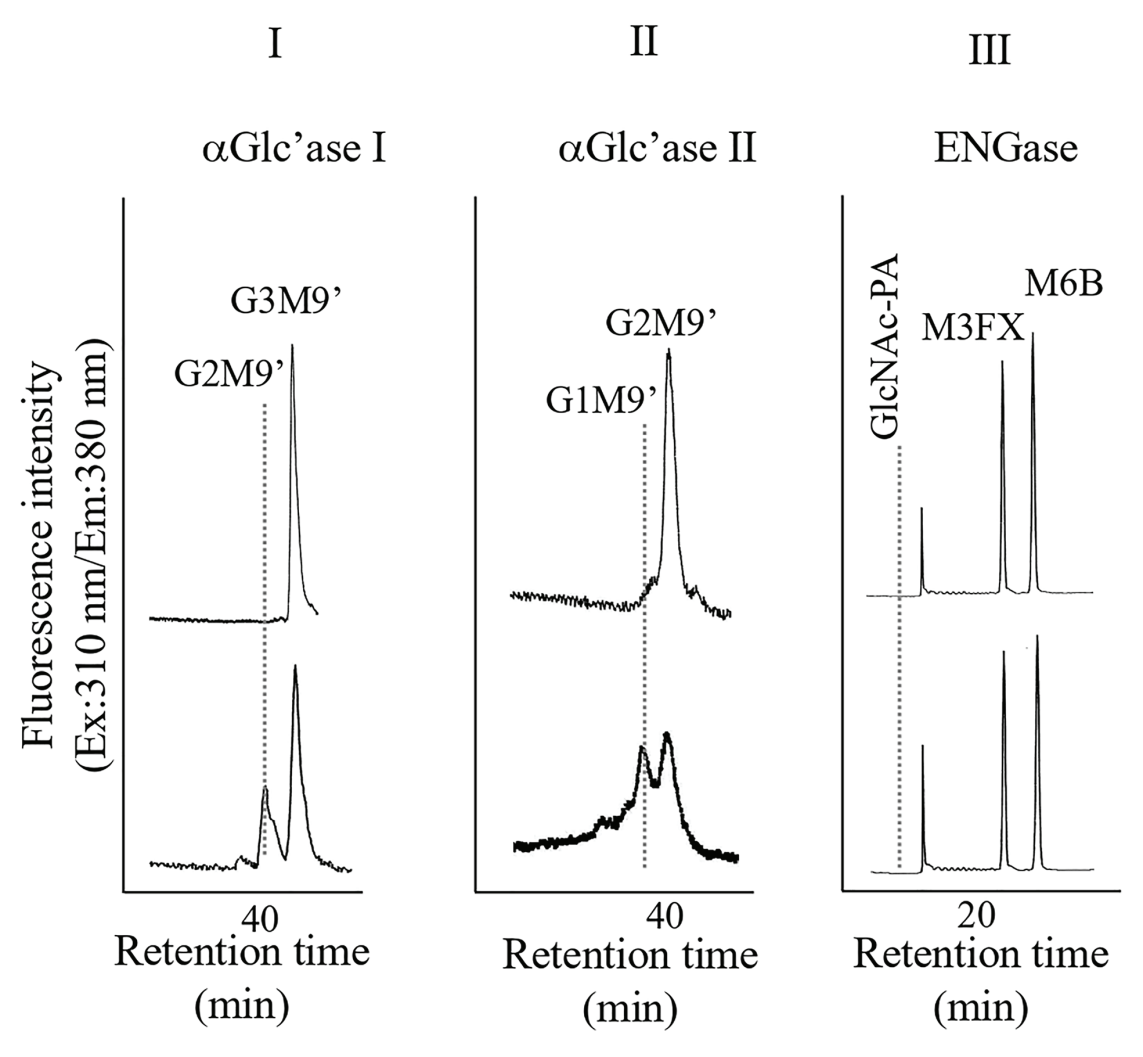
High performance liquid chromatography (HPLC) analyses of α-glucosidase I **(I)** and II **(II)** and endo-β-*N*-acetylglucosaminidase (ENGase; **III)** activities of the microsomes obtained from pumpkin hypocotyls α-Glc’ase I and II activities were assayed using Glc_3_Man_9_GlcNAc_1_-PA (G3M9’) and Glc2Man9GlcNAc1-PA (G2M9’), respectively, as substrates. The reaction mixtures were analyzed by size fractionation (SF)-HPLC, using a Shodex Asahipak NH2P-50 4E column. The ENGase activity was assayed using Man_6_GlcNAc_2_-PA (M6B) as a substrate and Man_3_Xyl_1_Fuc_1_GlcNAc_2_-PA (M3FX) as an internal standard. The reaction mixture was analyzed by reversed-phase (RP)-HPLC, using a Cosmosil 5C18-AR-II column.

Pumpkin (*Cucurubita* sp. cv. Kurokawa Amakuri) seeds (17.7 g) were soaked overnight, planted in moist rock fiber, and allowed to germinate at 25°C in the dark. The seedlings were grown for 6 days in the dark, and the etiolated hypocotyls were used for subcellular fractionation. Hypocotyls (41.5 g) were chopped using a razor and 0.15 M tricine buffer (50 ml, pH 7.5) containing 13% sucrose and 2 mM MgCl_2_ (buffer A). The chopped materials were squeezed through a nylon mesh. The resulting filtrate (10 ml each from 60 ml) was layered on the same tricine buffer, which consisted of two layers, a 20% sucrose layer (15 ml) and a 60% sucrose layer (10 ml), and centrifuged at 100,000 × *g* for 3 h (HITACHI 55P-72, Tokyo, Japan). After centrifugation, four fractions, from top to bottom (F-1, 10 ml; F-2, 10 ml; F-3, 10 ml; and F-IV, 5 ml), were collected, as shown in Supplementary Figure 1-I. Significant ENGase activity was detected in F-1 and F-2, indicating that these fractions contained cytosolic components (Supplementary Figure 1-II). On the contrary, α-Glc’ase I activities were detected in F-3 (Supplementary Figure 1-III); therefore, we used F-3 for further preparation of microsomes, which contained mainly ER. F-3 was diluted with Buffer A (35 ml) and centrifuged at 100,000 × *g* for 3 h. After centrifugation, the resulting precipitates were resuspended and washed in the 13% sucrose-containing buffer (25 ml), and the suspended sample was centrifuged again at 100,000 × *g* for 3 h. A part of the resulting precipitates were solubilized with 25 mM HEPES-NaOH buffer (1 ml, pH 7.5) containing 0.1% Triton X-100 by ultrasonication. As shown in Figure 1, the solubilized F-3 precipitates contained α-Glc’ase I and II but not ENGase, suggesting that F-3 contained the ER compartment.

### Preparation and Pyridylamination of FNGs

The microsome fraction was heated in boiling water for 3 min and then solubilized with 25 mM Tris-HCl buffer (pH 7.5) containing 0.2% SDS by ultrasonication for 15 min. The solubilized samples were desalted using Dowex 1 × 2 resins, and the run-through fraction was pooled and concentrated to approximately 10 ml. The run-though fraction was applied onto a Sephadex G-25 superfine column (2.7 × 33 cm) equilibrated with 0.1 N ammonium water. The oligosaccharide fractions (elution volumes from 61 to 115 ml) were pooled and concentrated to dryness using a rotary evaporator. The residue was suspended in distilled water (approximately 1 ml) and lyophilized. The lyophilized oligosaccharides were pyridylaminated (Natsuka and Hase, 1998). An excess amount of 2-aminopyridine was removed by gel filtration using a Sephadex G-25 Fine (1.8 × 40 cm) in 0.1 M NH_4_OH. The pyridylaminated oligosaccharides were monitored using a JASCO FP-8200 Fluorescence Spectrometer.

### Electrospray Ionization Mass Spectrometry

LC/MS and MS/MS analyses of PA-oligosaccharides were performed using an Agilent 6,500 series HPLC-Chip/QTOF-MS system equipped with a microwell-plate auto sampler (maintained at 10°C), capillary sample loading pump, nanopump, HPLC-Chip interface, and an Agilent 6,520 Q-TOF LC/MS, as described in our previous report (Maeda et al., 2017). A porous graphitized carbon (PGC)-Chip (Agilent Technologies) was used for separation of the PA-sugar chains.

## Results and Discussion

### Structural Analysis of FNGs in the Pumpkin Microsomes

**Figure 2:**
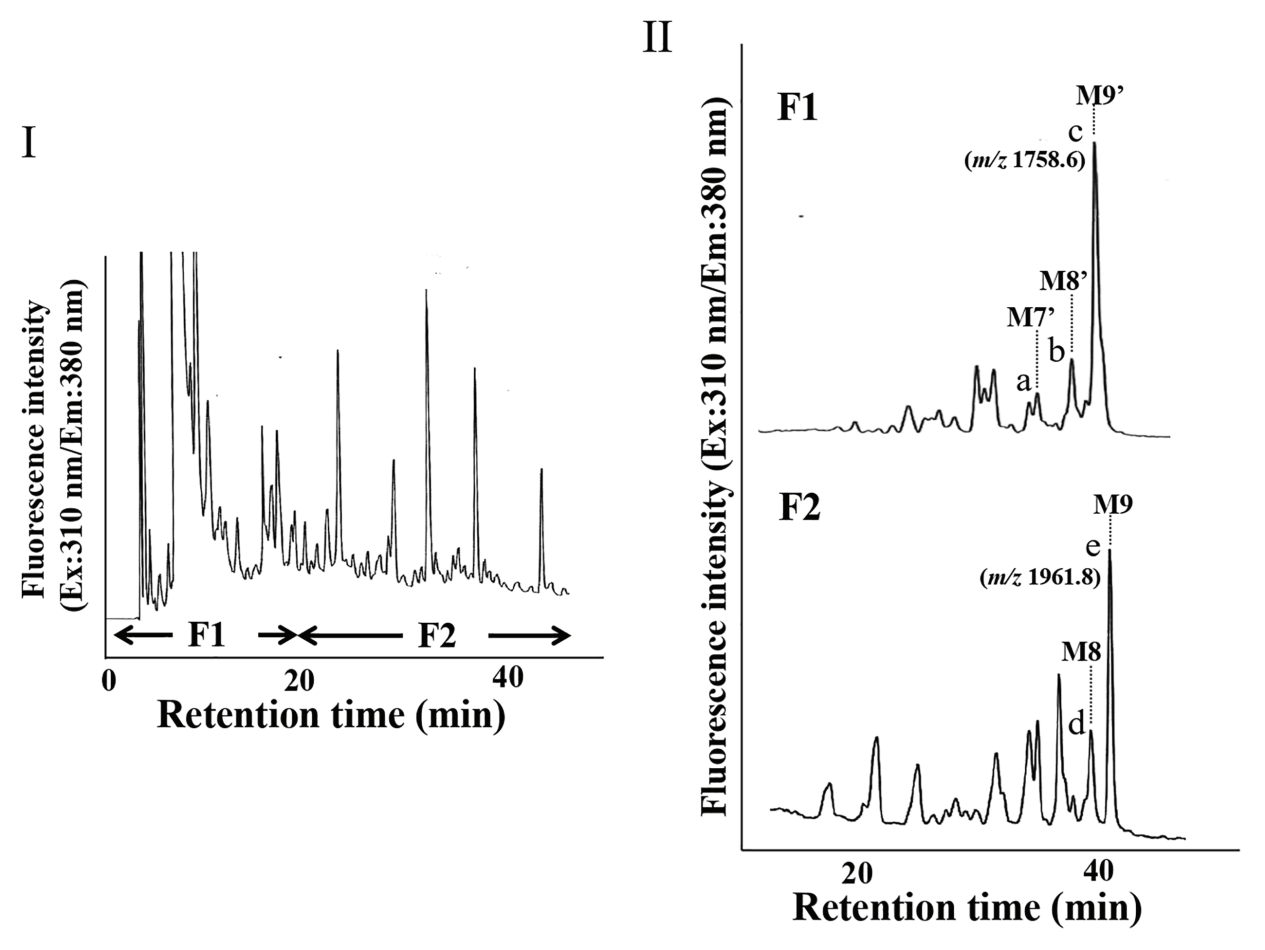
HPLC profiles of PA-oligosaccharides obtained from pumpkin microsomes. **(I)** The RP-HPLC profile of crude PA-oligosaccharides obtained from pumpkin microsomes is shown. The PA-oligosaccharides were separated by RP-HPLC using a Cosmosil 5C18-AR-II column. F1 contained GN1-FNGs, whereas F2 contained GN2-FNGs. **(II)** The SF-HPLC profiles of F1 and F2 obtained in **(I)**. M9’, M8’, and M7’ indicate the elution positions of authentic PA-oligosaccharides (Man_9_GlcNAc_1_-PA, Man_8_GlcNAc_1_-PA, and Man_7_GlcNAc_1_-PA, respectively). M9 and M8 indicate the elution positions of authentic PA-oligosaccharides (Man_9_GlcNAc_2_-PA and Man_8_GlcNAc_2_-PA, respectively).

**Figure 3:**
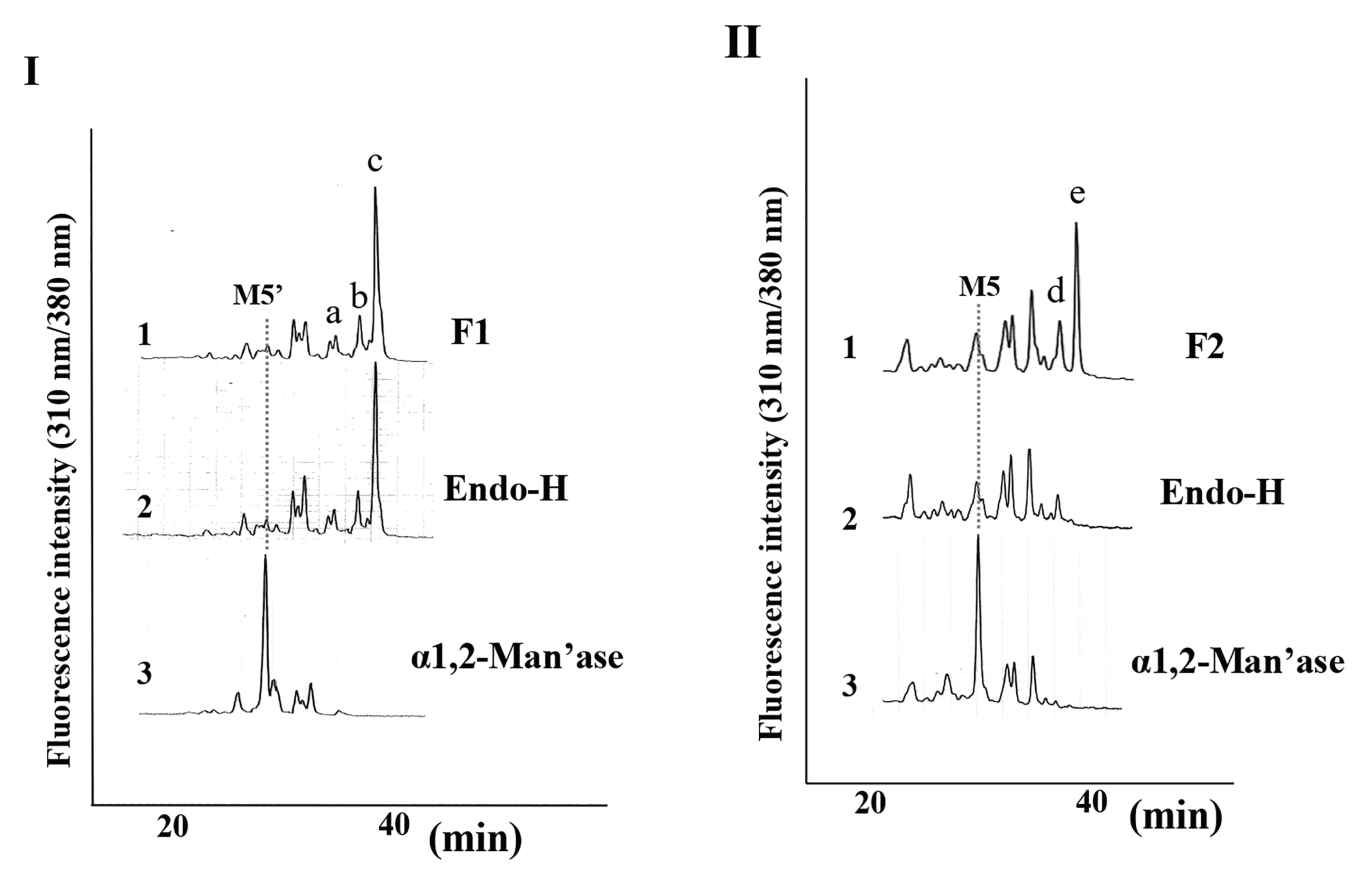
Structural analysis of free *N*-glycans (FNGs) in pumpkin microsomes. **(I)** SF-HPLC of glycosidase-digested F1. 1, F1 obtained in Figure 1. 2, End-H-digested F1. 3, α-1,2-Man’ase-digested F1. M5’ indicates the elution position of authentic Man_5_GlcNAc_1_-PA. **(II)** SF-HPLC of glycosidase-digested F2. 1, F2 obtained in Figure 1. 2, End-H-digested F1. 3, α-1,2-Man’ase-digested F1. M5 indicates the elution position of authentic Man_5_GlcNAc_2_-PA.

Since we confirmed that the microsomal fraction obtained from the pumpkin hypocotyls showed α-Glc’ase I and II activities, but not ENGase activity, as shown in Figure 1, we prepared pyridylaminated oligosaccharides from the 0.1% SDS-extract of the microsomes (mainly ER). First, the pyridylaminated oligosaccharides obtained from the microsomes were partially purified by RP-HPLC, as shown in Figure 2-I. As described in our previous reports (Kimura and Matsuo, 2000; Maeda et al., 2010, 2017), GN1-FNGs were eluted in the run-through or slightly retained fraction (F1, in this RP-HPLC system before 20 min), and GN2-FNGs were eluted in the bound fraction (F2, after 20 min). The PA-oligosaccharides in these two fractions were further analyzed by SF-HPLC. As shown in Figure 2-II, the elution positions of peaks a, b, and c obtained from F1 coincided with those of authentic M7’, M8’, and M9’, respectively, whereas the elution positions of peaks d and e from F2 coincided with those of authentic M8 and M9, respectively. Peaks c and e could be analyzed by electrospray ionization mass spectrometry (ESI-MS), as shown in Supplementary Figures 2, 3. For peak c, a parent ion was observed at *m/z* 879.8 as a double-charged ion, indicating that this signal was obtained from M9’ (Man_9_GlcNAc_1_-PA). All signals obtained from peak c by MS/MS analysis could be assigned to fragment ions from Man_9_GlcNAc_1_-PA. For peak e, a parent ion was observed at *m/z* 981.38 as a double-charged ion, indicating that this signal was obtained from M9 (Man_9_GlcNAc_2_-PA). All signals obtained from peak e by MS/MS analysis could be assigned to fragment ions from Man_9_GlcNAc_2_-PA. The structures of GN1- and GN2-FNGs in the pumpkin microsomes were further analyzed by exoglycosidase digestion. As shown in Figure 3-I, when the PA-oligosaccharides obtained from F1 and F2, as shown in Figure 2-II, were treated with Endo-H, peaks a, b, and c were not digested, but peaks d and e were digested as shown in Figure 3-II, indicating that peaks d and e represented HMT-GN2-FNGs. However, peaks a, b, and c were converted to M5’ (Man_5_GlcNAc_1_-PA) upon α-1,2-Man’ase digestion, indicating that peaks a, b, and c represent Man_7_GlcNAc_1_-PA, Man_8_GlcNAc_1_-PA, and Man_9_GlcNAc_1_-PA, respectively. The positions of peaks d and e were converted to M5 (Man_5_GlcNAc_2_-PA) upon α-1,2-Man’ase digestion, indicating that peaks d and e represented Ma_8_GlcNAc_2_-PA and Ma_9_GlcNAc_2_-PA, respectively. These results indicated that in addition to HMT-GN2-FNGs, HMT-GN1-FNGs (cytosolic ENGase products) occurred in the ER compartments. The yield of these pyridylaminated GN1-FNGs (M7’, M8’, and M9’) was approximately 40 pmol/g hypocotyls, whereas that of GN2-FNGs (M8 and M9) was approximately 30 pmol/g hypocotyls. The total amount of the HMT-GN1/GN2-FNGs obtained by the Con-A affinity chromatography from the soluble fraction (F-1 and F-2) was about 2.7 nmol/g hypocotyl, indicating that the amount of HMT-FNGs obtained from the microsomes was 1/40 of those in the soluble fraction. However, it seems that the amount maybe not reflect the real amount of FNGs occurring in the ER, since it is unlikely that total ERs have been collected during the fractionation process (some part of ERs maybe have been broken). At this moment, therefore, the real amount of FNGs occurring in the ER is obscure.

**Figure 4:**
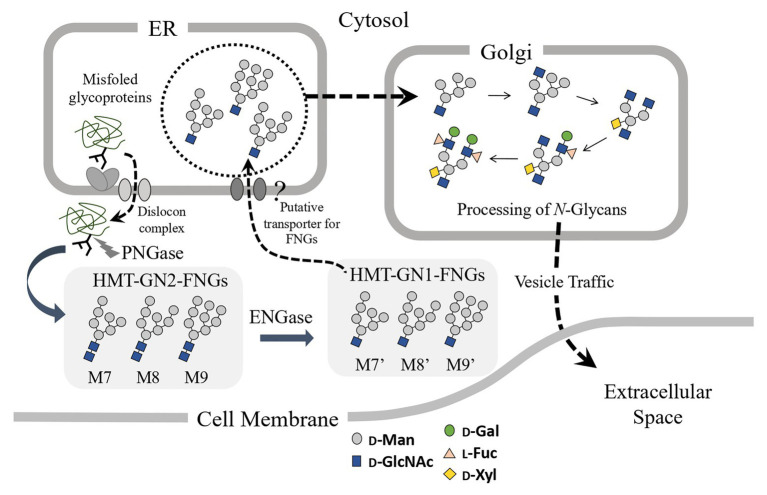
Schematic representation of the putative processing and secretion pathway of plant complex type (PCT)-GN1-FNGs based on the structural features of high-mannose type (HMT)-GN1-FNGs found in pumpkin microsomes is shown. The putative transporter for the HMT-GN1-FNGs produced from the HMT-GN2-FNGs in the cytosol by ENGase has not yet been found.

Since the microsomes prepared from the pumpkin hypocotyls showed α-Glc’ase I (a soluble enzyme) and α-Glc’ase II (a membrane-bound enzyme) activities, the microsomes probably contained intact ER compartments. The presence of HMT-GN1-FNGs (Man_9-7_GlcNAc_1_) in the ER compartments suggested that these HMT-GN1-FNGs produced in the cytosol were retro-transported to the ER from the cytosol through unidentified transporter(s) specific for these free oligosaccharides. If this was true, these HMT-GN1-FNGs, together with well-folded *N*-glycoproteins, might have been transported to the Golgi apparatus and processed into PCT-GN1-FNGs, as observed in previous reports (Maeda et al., 2010, 2017). Finally, PCT-GN1-FNGs were secreted into the extracellular space, as shown in Figure 4. It appeared that this hypothetical scheme could explain why PCT-GN1-FNGs, which contained the Lewis^a^ epitope [Galβ1-3(Fucα1-4)GlcNAcβ1-], were found in the culture broth, but not in the rice cells (Maeda et al., 2010). Additionally, it is possible that the microsomes prepared in this study contained the Golgi apparatus as a minor component and the HMT-FNGs were obtained from the Golgi apparatus. However, since the Golgi apparatus contains several kinds of α-Man’ases (Liebminger et al., 2009; Kajiura et al., 2010), the HMT-FNGs that occurred in the Golgi apparatus might have been trimmed into smaller *N*-glycans, such as Man_6-4_GlcNAc_1_, but not Man_9_GlcNAc_1_. In this study, such smaller size HMT-FNGs (Man_6-4_GlcNAc_1_) were not found, but in our previous study (Kimura et al., 2002), the very small amount of M5’ and M6’ in the ER-rich microsome fraction were found, which probably corresponded to F-3 in this study, and contamination of Golgi apparatus in F-3 cannot be completely excluded. Therefore, it seems to be necessary to assay the activities of Golgi-marker enzyme(s) to prove the complete absence or negligible amount of the Golgi apparatus in F3. This result will provide more solid evidence that the predominant occurrences of HMT-GN1-FNGs in the ER. In this study, we focused on HMT-FNGs in the ER, and at this moment it is obscure whether PCT-GN1-FNGs occur in the microsome fraction (F-3), although the amount might be very small if any. The structural analysis of FNGs in F-1 and F-2 is necessary for the next step to confirm whether the Golgi apparatus were mainly fractionated in F-2 but not F-3.

**Figure 5:**
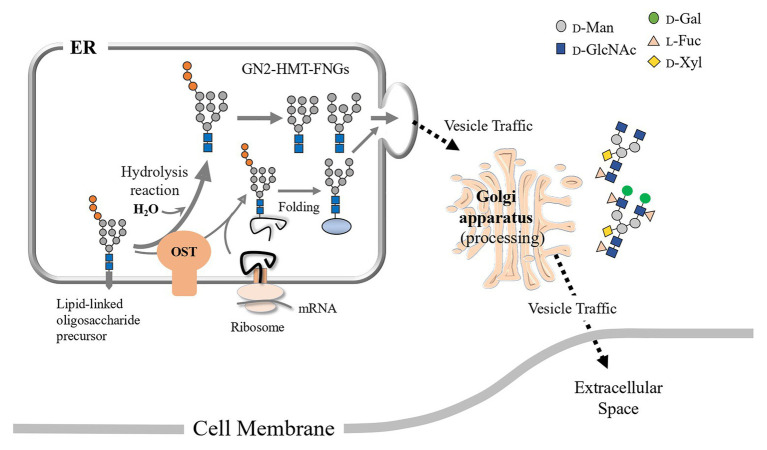
Schematic representation of the putative processing and secretion pathway of PCT-GN2-FNGs based on the structural features of HMT-GN2-FNGs found in pumpkin microsomes is shown. These PCT-GN2-FNGs have been found in soybean seedlings (Kimura and Kitahara, 2000), a freshwater plant (*Egeria densa*; Maeda et al., 2017), and the culture broth of rice cells (Maeda et al., 2010).

The HMT-GN2-FNGs, Man_9-8_GlcNAc_2,_ were also found in the pumpkin microsomes (or the ER compartment) in this study, suggesting that two putative mechanisms could be considered. One is that these GN2-FNGs were formed as byproducts during the transfer of Glc_1_Man_9_GlcNAc_2_ from the dolichol-oligosaccharide intermediates to the nascent polypeptides by OST (Harada et al., 2015), and the other is that, along with HMT-GN1-FNGs (ENGase products), the HMT-GN2-FNGs produced by cPNGase from misfolded glycoproteins were transported back to the ER. However, considering that the reaction rate of ENGase for HMT-GN2-FNG generation is very fast and the GN1-FNG concentration is greater than the GN2-FNG concentration (Kimura et al., 2002), the former mechanism appears to be more likely in plant cells, as shown in Figure 5. Furthermore, we recently confirmed that HMT-FNGs and PCT-FNGs occurred in a mutant line of *Arabidopsis thaliana*, in which one cPNGase and two ENGase genes were completely knocked out, indicating that HMT-GN2-FNGs were generated without cPNGase activity and converted into PCT-FNGs through the Golgi apparatus *via* a certain pathway (Shirai et al., 2019).

The putative transporter(s) responsible for the retro-transportation of HMT-GN1-FNGs from the cytosol to the ER have not yet been identified, and the identification of such glycan-specific transporter(s) is a prerequisite to evaluate our hypothesis or reveal the degradation mechanism of GN1-FNGs formed during plant ERAD.

## Data Availability Statement

The original contributions presented in the study are included in the article/Supplementary Material and further inquiries can be directed to the corresponding author.

## Author Contributions

YK, MK, NE, and MM wrote the manuscript. All authors contributed to the study concept and design and performed the study. All authors contributed to the critical revision of the manuscript.

### Conflict of Interest

The authors declare that the research was conducted in the absence of any commercial or financial relationships that could be construed as a potential conflict of interest.

## Figures and Tables

**Table 1:** 

TERM	Definition
ER	Endoplasmic reticulum
ERAD	ER-associated degradation
ENGase	Endo-β-*N*-acetylglucosaminidase
cPNGase	Cytosolic peptide:*N*-glycanase
OST	Oligosaccharyltransferase
α-Glc’ase I	α-Glucosidase I (ER α-1,2-glucosidase)
α-Glc’ase II	α-Glucosidase II (ER α-1,3-glucosidase)
α-1,2-Man’ase	α-1,2-Mannosidase
FNG	Free *N*-glycan
GN1-FNG	Free *N*-glycan bearing one GlcNAc residue at the reducing end
GN2-FNG	Free *N*-glycan bearing two GlcNAc residues at the reducing end
HMT-FNG	High-mannose type FNG
PCT-FNG	Plant complex type FNG
Le^a^	Lewis a
PA-	Pyridylamino
RP-HPLC	Reversed-phase HPLC
SF-HPLC	Size-fractionation HPLC
ESI-MS	Electrospray ionization mass spectrometry
Glc	D-Glucose
Man	D-Mannose
GlcNAc	*N*-acetyl-D-glucosamine
Xyl	D-Xylose
Fuc	L-Fucose
M6B	Manα1-6(Manα1-3)Manα1-6(Manα1-2Manα1-3)Manβ1-4GlcNAcβ1-4GlcNAc-PA
M3FX	Manα1-6(Manα1-3)(Xylβ1-2)Manβ1-4GlcNAcβ1-4(Fucα1-3)GlcNAc-PA
G3M9’	Manα1-2Manα1-6(Manα1-2Manα1-3)Manα1-6(Glcα1-2Glcα1-3Glcα1-3Manα1-2Manα1-2Manα1-3)Manβ1-4GlcNAc-PA
G2M9’	Manα1-2Manα1-6(Manα1-2Manα1-3)Manα1-6(Glcα1-3Glcα1-3Manα1-2Manα1-2Manα1-3)Manβ1-4GlcNAc-PA
Mn’	Man_n_GlcNAc_1_-PA
Mn	Man_n_GlcNAc_2_-PA

## Supplementary Material

Supplementary Figure 1 Preparation of microsomes mainly containing the ER compartments from pumpkin hypocotyls. **(I)** Composition of the sucrose-containing separation buffer used for microsome preparation by ultracentrifugation is shown. After ultracentrifugation (100,000 × *g* for 3 h), the samples were fractionated, as shown in the right figure. **(II)** HPLC analyses of the ENGase and α-Glc’ase I activities of the pumpkin microsomes obtained by the first ultracentrifugation were performed. α-Glc’ase I activity was assayed using Glc_3_Man_9_GlcNAc_1_-PA (G3M9’) as a substrate. The reaction mixtures were analyzed by SF-HPLC using a Shodex Asahipak NH2P-50 4E column. The ENGase activity was assayed using Man_6_GlcNAc_2_-PA (M6B) as a substrate and Man_3_Xyl_1_Fuc_1_GlcNAc_2_-PA (M3FX) as an internal standard. The reaction mixture was analyzed by RP-HPLC using a Cosmosil 5C18-AR-II column. Significant ENGase activity was found in F-1 in I, whereas significant α-Glc’ase I activity was found in F-3 in I.

Supplementary Figure 2 ESI-MS analysis of peak c obtained from F1 (as obtained in Figure 2-II). **(I)** ESI-MS analysis of peak c obtained in Figure 2-II. **(II)** MS/MS analysis of a signal at *m/z* 879.8 [M + 2H]^2+^.

Supplementary Figure 3 ESI-MS analysis of peak e obtained from F2 (as obtained in Figure 2-II). **(I)** ESI-MS analysis of peak e, as obtained in Figure 2-II. **(II)** MS/MS analysis of a signal at *m/z* 981.3 [M + 2H]^2+^.
