# Supplementary figures and images for: Cytosolic Free N-Glycans Are Retro-Transported Into the Endoplasmic Reticulum in Plant Cells
Source: Front Plant Sci. 2021 Jan 18;11:610124. doi: 10.3389/fpls.2020.610124 (PMC7847903; doi:10.3389/fpls.2020.610124)

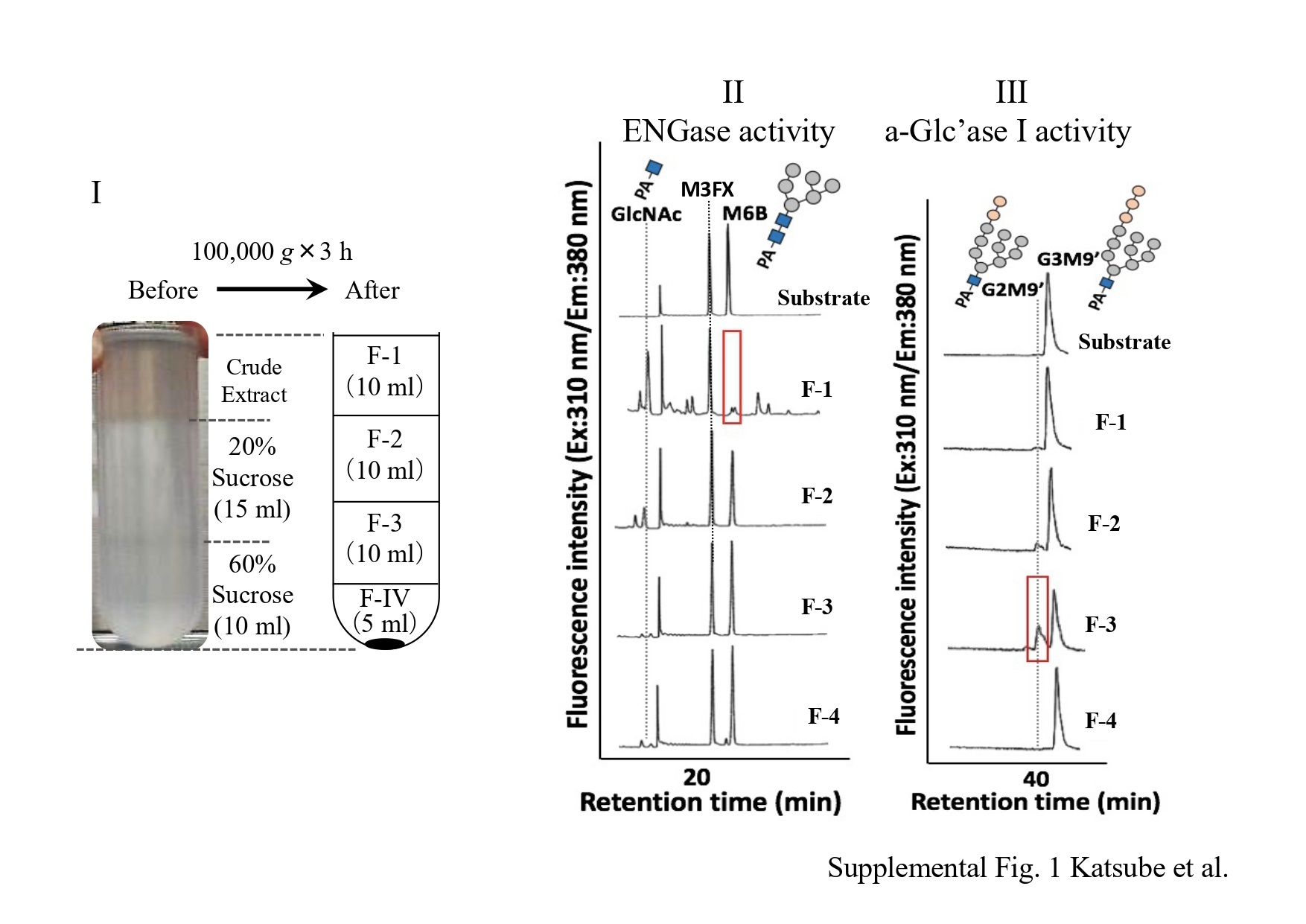

Supplement: Supplementary Figure 1 — Preparation of microsomes mainly containing the ER compartments from pumpkin hypocotyls. (I) Composition of the sucrose-containing separation buffer used for microsome preparation by ultracentrifugation is shown. After ultracentrifugation (100,000 × g for 3 h), the samples were fractionated, as shown in the right figure. (II) HPLC analyses of the ENGase and α-Glc’ase I activities of the pumpkin microsomes obtained by the first ultracentrifugation were performed. α-Glc’ase I activity was assayed using Glc3Man9GlcNAc1-PA (G3M9’) as a substrate. The reaction mixtures were analyzed by SF-HPLC using a Shodex Asahipak NH2P-50 4E column. The ENGase activity was assayed using Man6GlcNAc2-PA (M6B) as a substrate and Man3Xyl1Fuc1GlcNAc2-PA (M3FX) as an internal standard. The reaction mixture was analyzed by RP-HPLC using a Cosmosil 5C18-AR-II column. Significant ENGase activity was found in F-1 in I, whereas significant α-Glc’ase I activity was found in F-3 in I. [file Image_1.jpeg]

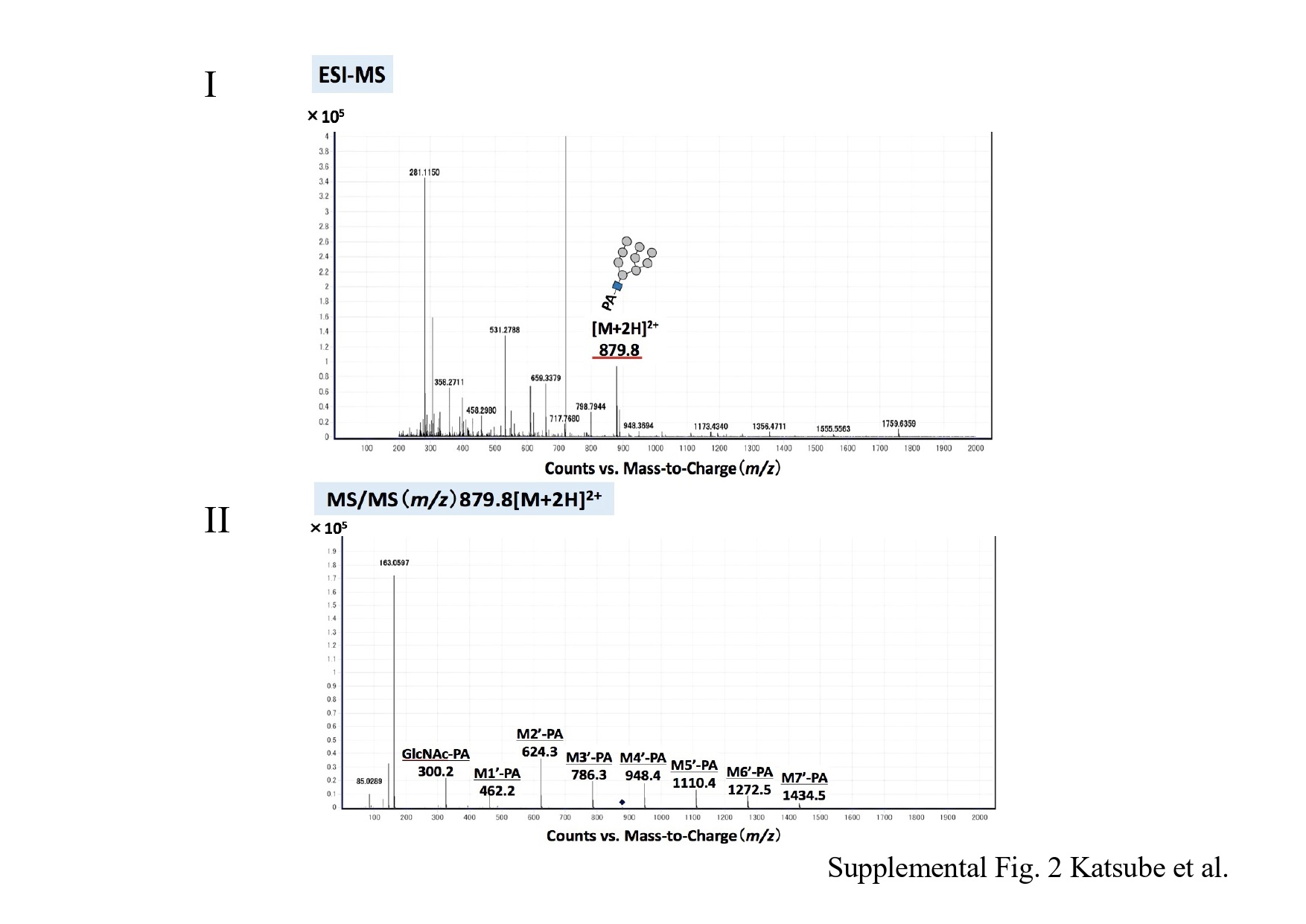

Supplement: Supplementary Figure 2 — ESI-MS analysis of peak c obtained from F1 (as obtained in Figure 2-II). (I) ESI-MS analysis of peak c obtained in Figure 2-II. (II) MS/MS analysis of a signal at m/z 879.8 [M + 2H]2+. [file Image_2.jpeg]

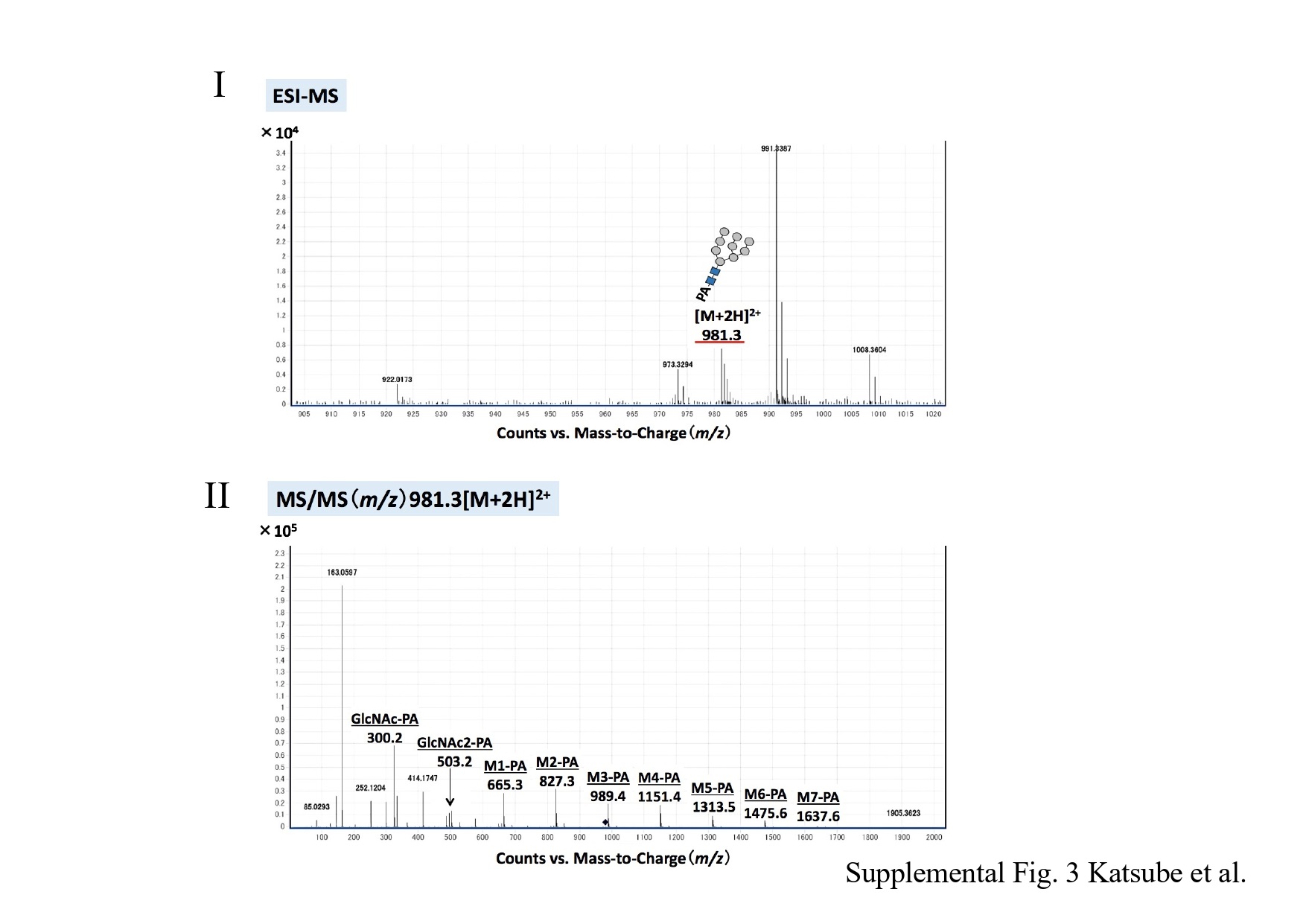

Supplement: Supplementary Figure 3 — ESI-MS analysis of peak e obtained from F2 (as obtained in Figure 2-II). (I) ESI-MS analysis of peak e, as obtained in Figure 2-II. (II) MS/MS analysis of a signal at m/z 981.3 [M + 2H]2+. [file Image_3.jpeg]
